# Neural Network Connectivity During Post-encoding Rest: Linking Episodic Memory Encoding and Retrieval

**DOI:** 10.3389/fnhum.2018.00528

**Published:** 2019-01-09

**Authors:** Okka J. Risius, Oezguer A. Onur, Julian Dronse, Boris von Reutern, Nils Richter, Gereon R. Fink, Juraj Kukolja

**Affiliations:** ^1^Cognitive Neuroscience, Institute of Neuroscience and Medicine (INM-3), Research Centre Jülich, Jülich, Germany; ^2^Department of Neurology, University Hospital Cologne, Cologne, Germany; ^3^Department of Neurology and Neurophysiology, Helios University Hospital Wuppertal, Wuppertal, Germany

**Keywords:** functional connectivity, episodic memory consolidation, memory interference, memory trace, resting state fMRI

## Abstract

Commonly, a switch between networks mediating memory encoding and those mediating retrieval is observed. This may not only be due to differential involvement of neural resources due to distinct cognitive processes but could also reflect the formation of new memory traces and their dynamic change during consolidation. We used resting state fMRI to measure functional connectivity (FC) changes during post-encoding rest, hypothesizing that during this phase, new functional connections between encoding- and retrieval-related regions are created. Interfering and reminding tasks served as experimental modulators to corroborate that the observed FC differences indeed reflect changes specific to post-encoding rest. The right inferior occipital and fusiform gyri (active during encoding) showed increased FC with the left inferior frontal gyrus and the left middle temporal gyrus (MTG) during post-encoding rest. Importantly, the left MTG subsequently also mediated successful retrieval. This finding might reflect the formation of functional connections between encoding- and retrieval-related regions during undisturbed post-encoding rest. These connections were vulnerable to experimental modulation: Cognitive interference disrupted FC changes during post-encoding rest resulting in poorer memory performance. The presentation of reminders also inhibited FC increases but without affecting memory performance. Our results contribute to a better understanding of the mechanisms by which post-encoding rest bridges the gap between encoding- and retrieval-related networks.

## Introduction

After encoding, newly learned information is consolidated and stored into long-term memory (Lechner et al., 1999; Dudai, 2004). The precise neural mechanisms underlying this transformation to date remain poorly understood. Initial memory consolidation rapidly starts at the synaptic level, followed by system-level consolidation – a slower process which involves structural and functional modifications in large scale neural networks (Dudai, 2004). The most common theories of system-level consolidation propose that memory consolidation depends upon initial hippocampal encoding and a subsequent transfer of memory representations to neocortical regions (Squire and Alvarez, 1995; Bontempi et al., 1999; Frankland et al., 2001; Wiltgen et al., 2004; Smith and Squire, 2009). Beside the hippocampal formation, recent functional imaging data suggest that also neocortical regions, which mediate memory encoding, also remain active during post-encoding off-line consolidation (Foster and Wilson, 2006; Peigneux et al., 2006; Tambini et al., 2010; Kukolja et al., 2016). This activity may at least in part be due to repetitive (re-)activation of memory-specific neocortical networks, which seems to be one crucial process involved in early memory consolidation (Hoffman and McNaughton, 2002; Deuker et al., 2013; Atherton et al., 2015).

Commonly, a switch between networks mediating memory encoding and those mediating retrieval is observed (Nyberg et al., 1996; Lepage et al., 1998; Kukolja et al., 2009b; Spaniol et al., 2009; Huijbers et al., 2012). This switch may be due to different task demands during encoding and retrieval. Encoding requires, for example, the selection and organization of incoming information (Paller and Wagner, 2002; Simons and Spiers, 2003; Blumenfeld and Ranganath, 2007), whereas retrieval involves processes like matching incoming information with stored memories and post-retrieval monitoring and verification (Fletcher and Henson, 2001; Petrides, 2002; Simons and Spiers, 2003; Dobbins and Han, 2006; Burgess et al., 2007). On the other hand, these changes may at least in part reflect the formation of new neocortical memory traces and their dynamic change over time (Wang and Morris, 2010). Which of the involved networks are responsible for the creation of memory traces and how the latter are created during consolidation remains to be elucidated. In the present study, we therefore focused on post-encoding processes to gain a better understanding of the neural mechanisms underlying the formation of new links between encoding- and retrieval-related areas.

In a data-driven approach, we first determined regions mediating memory encoding and subsequent retrieval using task-related fMRI. The encoding-related regions then served as seeds for connectivity analyses during post-encoding rest measured by resting state functional MRI (rs-MRI).

Since the specificity of connectivity changes during rs-MRI is not readily evident, we introduced an experimental manipulation in order to challenge post-encoding processes both at the behavioral and neural level. To this end, we used interfering and reminding stimuli to interact with consolidation.

We hypothesized that during post-encoding rest, functional links between encoding- and retrieval-related regions are established but that these links are initially unstable and can hence be modified or disrupted by new memory contents. Furthermore, we expected interfering but not reminding stimuli to result in poorer memory performance, as the reminding stimuli did not provide competing information. With regard to consolidation processes, we expected that in particular interfering stimuli would modulate functional connectivity.

## Materials and Methods

### Participants

18 Participants (9 female, within the age range of 18–30 years, mean age 24.61, *SD* = 3.26) without prior history of neurological or psychiatric disease participated in this study. All of them were native German speakers, had normal or corrected to normal visual acuity, and were right-handed, as assessed by the Edinburgh handedness inventory (Oldfield, 1971). Informed written consent was obtained from all participants prior to examination. The study had been approved by the local ethics committee.

Participants underwent a detailed neuropsychological assessment including tests and questionnaires allowing the evaluation of their cognitive profile with regard to verbal episodic memory [VLMT, Verbaler Lern- und Merkfähigkeitstest (Helmstaedter and Lux, 2001)], visual spatial episodic memory [design memory task of the Wechsler Memory Scale (WMS) (Petermann and Lepach, 2012)], verbal short-term and working memory [Digit Span of the WMS (Petermann and Lepach, 2012)], visual working memory [Symbol Span of the WMS (Petermann and Lepach, 2012)], psychomotor speed [TMT-A, Trail Making Test, part A (Bowie and Harvey, 2006)], executive functioning [TMT-B, Trail Making Test, part B (Bowie and Harvey, 2006) and Stroop Test (Stroop, 1935)], mental rotation [LPS 7, Leistungsprüfsystem (Horn, 1983)], attention [BTA, Brief Test of Attention (Schretlen, 1997)], word fluency [RWT, Regensburger Wortflüssigkeits-Test (Aschenbrenner et al., 2000)], and premorbid intelligence [MWT-B, Mehrfachwahl-Wortschatz-Intelligenztest (Lehrl, 2005)]. Additionally, a semi-structured interview was used to survey depressive and psychiatric symptoms [Hamilton depression scale, HAM-D (Hamilton, 1960)]. Demographic data were obtained to control for inter-individual differences, such as the educational level. In sum, all participants were free of neuropsychological impairment or psychopathology.

### Experimental Design

Each participant completed three fMRI sessions approximately 1 week apart [mean 8.31 days, standard error (SE) 0.43]. During each session, participants performed a memory task comprised of an encoding and a retrieval run (durations 8.0 min and 6.4 min, respectively). During encoding and retrieval, task-related fMRI was measured in order to define regions of interest for the analysis of rs-fMRI sessions. Further details will be described in section “Statistical Analysis of Task-Related fMRI Data.”

Additionally, rs-fMRI measurements were conducted at three time points during each session: at baseline, i.e., before encoding (R1), during early rest after encoding (R2), and during delayed rest before retrieval (R3), with a duration of 7.0 min each. During rest, participants were instructed to relax with eyes closed without falling asleep.

The three sessions differed with regard to the nature of a modulatory task introduced during post-encoding rest. Between rest periods R2 and R3, a task (duration 8.0 min) was introduced in order to interfere with potential consolidation. Depending on the session, the task contained either interfering stimuli, reminders, or neutral stimuli (see below for details).

### Task

Stimuli were presented on an LCD (liquid crystal display) screen. For stimulus delivery and response recording, the program Presentation (Neurobehavioral Systems, Inc., Albany, CA, United States) was used. Participants responded by pressing buttons on a Lumitouch keyboard, which was placed under the participant’s right hand.

**Figure 1:**
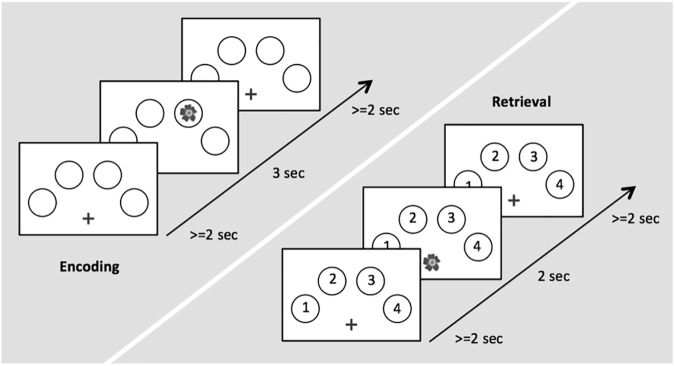
Time course of stimulus presentation for the encoding (left) and the retrieval session (right).

During task-related fMRI, participants performed an adaptation of a well-established memory task providing object-location associations to be remembered (Cansino et al., 2002; Kukolja et al., 2009a,b). The task consisted of an encoding and a retrieval session. Stimuli were color photographs of 32 natural and 32 artificial (man-made) objects. To counter-balance stimuli across subjects and conditions, three different sets of 64 pictures were created. The sets were assigned randomly to subject and scanning session so that no set of pictures was used twice for different experimental conditions. Four circles were displayed on a white screen, arranged in a horizontal semi circle bent to the top (Figure 1). During encoding, the 64 objects were presented randomly in one of the four circles. Each picture was presented for 3.0 s, with an inter-stimulus interval (ISI) of 2.0–12.0 s. This variable ISI allowed the BOLD signal to be captured at different time points after stimulus presentation in order to obtain a better representation of its time course. The participants were asked to memorize the respective position at which each stimulus appeared. To ensure that the participants constantly paid attention, participants were instructed to indicate via button press with the index of their right hand if the object was natural and with the middle finger of their right hand if the object was artificial. During retrieval, each previously shown picture was presented again at the center of the screen. Each picture was presented for 2.0 s, with an ISI of 2.0–12.0 s. Participants were asked to indicate via button press with the four fingers of their right hand in which location the object had appeared during the encoding run. Thus, we were able to differentiate between successfully and erroneously encoded and retrieved object-location associations. The trial designs of encoding and retrieval sessions are illustrated in Figure 1.

**Figure 2:**
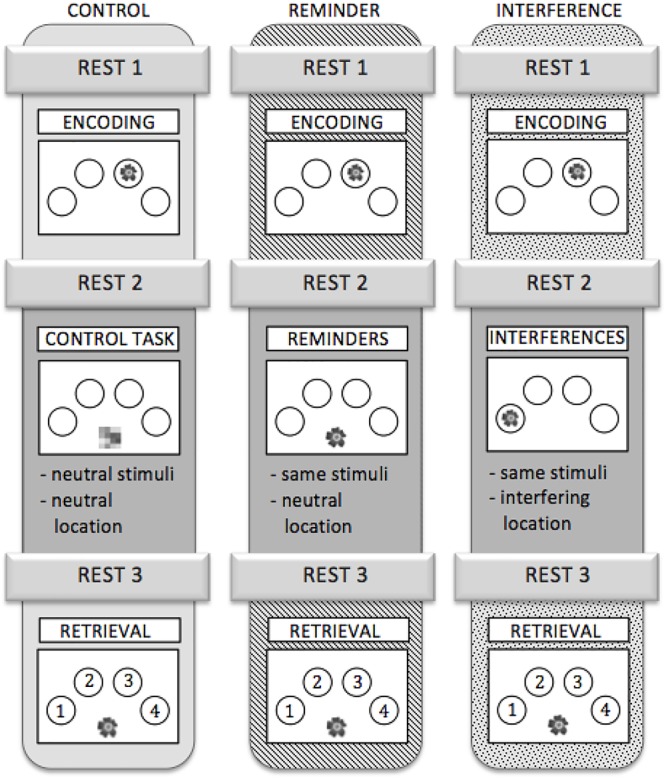
Outline of the three scanning sessions. Each participant completed all three sessions. The sessions were assigned to the subjects in a randomized order.

To test whether connectivity changes during post-encoding rest could be attributed to consolidation of previously learned material, we added a cognitive intervention during encoding and retrieval, i.e., between resting state fMRI runs R2 and R3. Three different types of intervention were used: (1) an interference task (IN), (2) a task which provided reminders for previously learned object-location association (RE), or (3) a control task (C) (Figure 2).

IN: All objects, which had been shown during the encoding run, were presented again but at different locations than during encoding. Participants were asked to ignore the interfering information. Analogous to the encoding run, participants were instructed to make natural-artificial judgments for the objects presented (Figure 2).

RE: During this task, all stimuli that had been presented in the encoding run re-appeared, but were now presented in the midline at the bottom of the screen. Stimuli served as a cue for (implicit) reactivation of the memory for their respective location during the preceding encoding run. Again, the participants were asked to decide whether the objects were natural or artificial (Figure 2).

C: During the control task, abstract pictures without any informational content were presented in the midline at the bottom of the screen. Participants were asked to press a button with their index finger each time a picture appeared on the screen (Figure 2). The control task neither provided interference nor any kind of reminder, while variables like motor activity and visual input were comparable to the other conditions.

Stimulus on times during IN, RE, and C were equal and corresponded to those during encoding. Different sets of stimuli were used for the three different sessions. To control for sequence and training effects, the different versions were assigned to three fMRI scanning sessions in a randomized order across participants. A detailed schedule for the experiment is depicted in Figure 2.

### Post-scanning Questionnaires

At the end of each scanning session, subjective tiredness was surveyed retrospectively for the three resting states using a 6-point-scaled questionnaire. Additionally, participants were asked to report whether they had fallen asleep. Concerning the memory task, participants were asked whether or not they deliberately rehearsed the presented material during task-free sessions. Unanimously, participants negated an active rehearsal of the information.

### fMRI Data

fMRI data acquisition. Whole-brain imaging was conducted using a 3 Tesla whole body MRI system, equipped with a 12-channel phase array head coil (Siemens, Magnetom Trio, Erlangen, Germany). T2^∗^-weighted echoplanar images with blood oxygen level dependent (BOLD) contrast were obtained with the following parameters: Repetition time (TR) = 2.43 s, echo time (TE) = 30 ms, matrix size = 64 × 64, pixel size = 3.1 mm^2^ × 3.1 mm^2^, slice thickness = 3 mm, flip angle = 90°, distance factor = 10%, field of view = 200 mm, 40 axial slices, acquired volume number = 180 (resting state) and 195–240 (task-related measurements across the different conditions; *n* = 240 scans were acquired during encoding, *n* = 195 scans were acquired during retrieval, and *n* = 220 scans were acquired during IN, RE, and C runs), covering the participant’s whole brain.

Moreover, a high-resolution anatomical image was acquired using a three-dimensional magnetization-prepared, rapid acquisition gradient echo (MP-RAGE) sequence (details: 1 mm x 1 mm x 1 mm).

#### Image Processing

Image processing and statistical analyses were performed using MATLAB-based toolboxes (R2014a, Mathworks, Sherborn, MA, United States). Preprocessing was carried out with the toolbox DPARSF (Data Processing Assistant for Resting-State fMRI) version 3.2 (^1^Yan and Zang, 2010) which is based on SPM8 (statistical parametric mapping software, Wellcome Department of Imaging Neuroscience, London, United Kingdom^2^) and DPABI (toolbox for Data Processing & Analysis of Brain Imaging^3^). Preprocessing steps included segmentation of structural T1 images into gray matter (GM), white matter (WM), and cerebrospinal fluid, coregistration to functional images, followed by normalization to MNI (Montreal Neurological Institute) space using DARTEL (Diffeomorphic Anatomical Registration Through the Exponentiated Lie algebra) technique. A 6 mm FWHM (full-width at half-maximum) Gaussian kernel was used for spatial smoothing. Furthermore, functional images were realigned and normalized to the MNI space with a resampling voxel size of 3 mm × 3 mm × 3 mm.

Depending on the length of the instructions shown at the beginning of each fMRI run, the first volumes during which MR signal equilibrium was reached (encoding: *n* = 18, retrieval *n* = 13, R1, R2, R3: *n* = 7, IN: *n* = 16, RE: *n* = 13, C: *n* = 13) were discarded, during which participants also adapted to the MR scanning noise and read task instructions.

#### Statistical Analysis of Task-Related fMRI Data

Although the focus of the analyses was on post-encoding rest, we first analyzed task-related fMRI data in order to define study-specific regions correlating with successful encoding. The encoding-related clusters then served as seeds for connectivity analyses of the resting-state fMRI data (see below) since we expected that memory-related activity and hence connectivity would originate in these areas and persist during post-encoding rest.

Secondly, we analyzed task-related fMRI data during retrieval in order to determine whether target clusters showing increased connectivity with encoding-related clusters during post-encoding rest (see above) would correspond to or overlap with retrieval-related clusters.

Task-related fMRI data of the encoding and retrieval runs were analyzed in an event-related fashion. Encoding data were analyzed by defining two effects of interest (subsequently remembered object-location associations, E_C, and subsequently forgotten object-location associations, E_F) at the first level. Analogously, two effects of interest (correctly remembered object-location associations, R_C, and forgotten object-location associations, R_F) were defined at the first level during retrieval. Each experimental condition was modeled using a boxcar reference vector convolved with a canonical hemodynamic response function and its first-order temporal derivative. The boxcar length for each event was determined by the corresponding stimulus duration in order to account for visual processing time. Parameter estimates were subsequently calculated for each voxel using weighted least squares to provide maximum likelihood estimators (Kiebel and Holmes, 2003). No global scaling was applied. The parameter estimates for the HRF and linear contrasts of these estimates comprised the data for the second level analysis.

At the second level, paired *t*-tests were performed. For encoding, the subsequent memory effect (SME) was defined as the difference between activity during trials with subsequently remembered object-location associations and activity during trials where object-location associations were subsequently forgotten (E_C > E_F). Specific activity mediating successful retrieval of object-location associations was defined by calculating the difference between activity associated with correctly remembered and forgotten object-location associations (R_C > R_F).

Data of eighteen participants were included in the analyses of the encoding runs. Only data of sixteen participants entered the analyses of retrieval data since two participants had to be excluded due to excessive head movements during the retrieval run (translation of >3 mm in any direction (*x*, *y*, *z*) or rotation of >3 degrees).

We first performed exploratory analyses to find regions generally involved in encoding processes using a more lenient threshold of *p* < 0.001, uncorrected for multiple comparisons. For the seed based analyses of post-encoding neural activity we chose clusters whose peak survived a threshold of p-FWE < 0.05, to ensure low likelihood of false positives.

#### Statistical Analysis of Resting State fMRI Data

Rs-fMRI was performed to investigate post-encoding neural processes by examining FC during rest. FC is a measure of the temporal correlation between spatially separated neurophysiological processes (Friston, 2011). Analyses were carried out with the functional connectivity toolbox CONN (^4^Whitfield-Gabrieli and Nieto-Castanon, 2012).

We calculated temporal bivariate correlations between the blood oxygenation level-dependent (BOLD) signal from a specified region of interest (ROI) to all other voxels in the brain (seed-to-voxel analyses) as well as between two specified ROIs (ROI-to-ROI analyses).

The component-based noise correction method implemented in the toolbox was used to reduce potential confounds due to head movement or non-gray matter tissue signal. To this end, white matter signal, cerebrospinal fluid signal, and realignment parameters were entered as nuisance variables (Behzadi et al., 2007), substantially increasing the sensitivity and reliability of functional connectivity analyses (Whitfield-Gabrieli and Nieto-Castanon, 2012). Moreover, scrubbing was applied to remove signal peaks due to head movements using a frame-wise displacement (FD) threshold of 0.5. The data were band-pass filtered to 0.008–0.09 Hz.

Regions associated with the SME during encoding with p-FWE < 0.05 served as spatial templates for the ROIs for the seed-to-voxel analyses. To increase power, the SME was calculated across all three experimental conditions. FC analyses resulted in spatial maps consisting of fisher-transformed correlation coefficients.

Since we were primarily interested in post-encoding FC after cognitive modulation or control, we focused our analysis on the resting state run preceding retrieval (R3) and compared it to baseline (R1).

In a stepwise procedure, we first performed seed-to-voxel analyses on resting state sessions of the control condition in order to find neural changes unique to undisturbed post-encoding rest compared to baseline. As specified above, encoding-related ROIs were used as seeds, since we expected post-encoding activity and connectivity to originate in these areas. Retrieval-related ROIs were not used as seeds in this analysis since this would violate the putative temporal order of connectivity changes.

In order to define which FC changes were specific to post-encoding neural processes, we calculated paired *t*-tests comparing seed-to-voxel correlation values of R1 with those of R3. The individual difference in subjective tiredness values between the two resting states (Δ fatigueR3 – fatigueR1) was included as a nuisance covariate.

The seed-to-voxel analyses yielded target clusters showing significant connectivity changes with the seed regions during post-encoding rest.

In order to determine whether modulation (IN, RE, when compared to C) changed this seed to target connectivity, the seeds and target clusters were subjected to ROI-to-ROI-analyses in a second step. For this, ANOVAS were performed with the factors modulation (IN, RE, C) and resting state [baseline (R1), post-encoding rest (R3)] for the three different ROI-combinations. The primary objective of this ANOVA was to detect interaction effects between the factors modulation and resting state. *Post hoc t*-tests were then applied to further determine the influence of modulation.

As we were especially interested in changes of FC from baseline (R1) to post-encoding rest (R3), we calculated the difference between FC values of the two resting states (Δ FCR3 – FCR1) in order to compare the extent of FC changes across the experimental conditions.via *post hoc t*-tests.

### Statistical Analysis of Behavioral Data

Behavioral data collected during the fMRI-paradigm were analyzed using IBM SPSS Statistics^®^ (version 23, IBM Corp., Armonk, NY, United States). Memory performance was represented by the number of correctly retrieved object-location-associations. One sample *t*-tests were performed to confirm that correct responses within each experimental condition were not produced by chance. Differences between the conditions were analyzed by use of a repeated measures ANOVA with condition (IN, RE, C) as a within-subject factor. In addition, we analyzed the interference condition separately focusing on the nature of false location judgments during retrieval. Two types of mistakes could be distinguished: Intrusions, defined as trials where the object location of the interference run rather than the location of the encoding run was retrieved, and general mistakes, defined as trials where the judgment neither corresponded to object location during the interference run nor the encoding run.

A one sample *t*-test was performed to test whether the number of intrusions significantly differed from chance.

## Results

### Behavioral Results

#### Memory Performance

**Figure 3:**
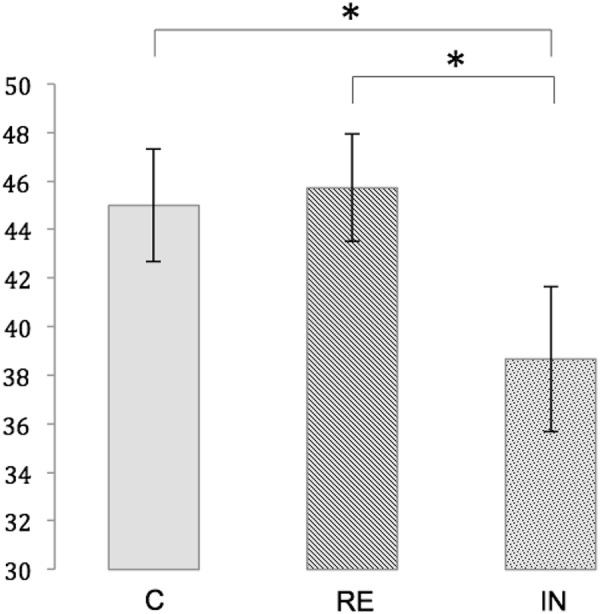
Memory performance for the conditions C (control), RE (reminder) and IN (interference). Depicted is the mean number of correct responses with standard errors. Significant differences at *p* < 0.05 are marked with an asterisk.

Within each experimental condition, the number of correct responses significantly differed from chance, as demonstrated by one sample *t*-tests (test score: 16; mean number of correct responses [standard error (SE)]: IN: 38.67 (3.01), *t*(17) = 7,528, *p* < 0.01, RE: 45.72 (2.20), *t*(17) = 13.517, *p* < 0.01, C: 45.00 (2.33), *t*(17) = 12.421, *p* < 0.01. A repeated measures ANOVA showed a significant main effect for memory performance [*F*(2,34) = 6.330, *p* < 0.01]. *Post hoc t*-tests revealed a significantly lower number of correct responses in the interference condition compared to both the reminder condition [*t*(17) = -2.724, *p* < 0.05, Bonferroni-corrected] and the control condition [45.00 (2.33), *t*(17) = -2.892, *p* < 0.05, Bonferroni-corrected]. Memory performance was not significantly different between the reminder condition and the control condition [*t*(17) = 0.433, *p* = 1.00, Bonferroni-corrected]. Figure 3 illustrates the memory performance for the three conditions.

#### Intrusions

In the interference condition, 47.69% of the total number of mistakes was classified as intrusions. Considering that the chance probability for this kind of mistake among all possible wrong responses was about 1/3, the percentage of intrusions was significantly different from chance (test score: 33.33%, percent mean (SE) of intrusions: 47.69% (3.58), [*t*(17) = 4.008, *p* < 0.01].

### Fatigue Scores

A repeated measures ANOVA was performed on the subjective fatigue scores with the within-subject factors condition (IN, RE, C) and rest (R1, R3).

The main effect of rest revealed a non-significant trend for an increase of subjective tiredness from the first to the third resting state run [mean (SE)]: R1: 2.67 (0.21), R3: 3.15 (0.19), [*F*(1,17) = 4.244, *p* = 0.055]. There was no effect of condition on fatigue scores [mean (SE)]: IN: 2.78 (0.20), RE: 3.11 (0.26), C: 2.83 (0.20), [*F*(2,34) = 0.907, *p* = 0.413] and no interaction between condition and rest [*F*(2,34) = 0.855, *p* = 0.434].

### Task-Related fMRI Data

**Figure 4:**
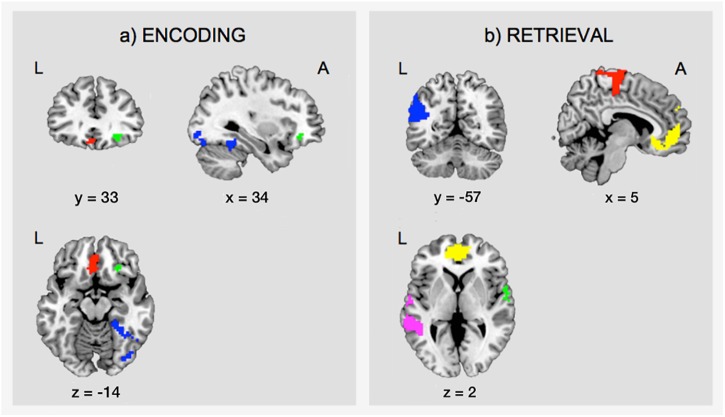
Task-related fMRI data. The figure shows **(a)** the subsequent memory effect during encoding (green, orbitofrontal cortex; blue, right inferior occipital gyrus and fusiform gyrus; red, left ventromedial prefrontal cortex), and **(b)** the main effect of memory during retrieval (yellow, ventromedial prefrontal cortex; pink, middle temporal gyrus; green, superior temporal gyrus; blue, angular gyrus; red, precentral gyrus). The figure shows clusters whose peak survived a threshold of p-FWE < 0.05. For display purposes, clusters are depicted at a threshold of *p* < 0.001, uncorrected for multiple comparisons. L, left; A, anterior.

During encoding, an SME (E_C > E_F) was found in a distributed network including the inferior frontal gyrus (IFG) bilaterally, the hippocampal formation bilaterally, the left fusiform gyrus, the left inferior parietal lobule, the left lingual gyrus, and the right inferior occipital gyrus (IOG)/fusiform gyrus (Table 1A and Figure 4a).

**Table 1:** Task-related fMRI data during encoding and retrieval.

		*x*	*y*	*z*	*Z*	Peak p-FWE	Voxels
**(A) Main effect of memory during encoding (E_C > E_F)**							

**orbitofrontal cortex**	**R**	**27**	**33**	–**12**	**5.00**	**0.009**	**30**
**IOG/fusiform gyrus**	**R**	**45**	–**75**	–**12**	**4.86**	**0.017**	**238**
**vmPFC**	**L**	–**3**	**33**	–**18**	**4.65**	**0.042**	**55**
Fusiform gyrus	L	–45	–48	–18	4.59	0.053	202
IFG (orbital)	L	–33	30	–3	4.35	0.128	69
Fusiform gyrus	L	–27	–39	–18	4.28	0.164	34
IFG (triangular)	R	51	39	15	4.05	0.337	28
Lingual gyrus	L	–18	–93	–15	3.91	0.482	110
Inferior parietal lobule	L	–51	–72	30	3.89	0.504	18
Hippocampal formation	R	24	–24	–12	3.76	0.658	12
Hippocampal formation	L	–21	–6	–18	3.69	0.734	20

**(B) Main effect of memory during retrieval (R_C > R_F)**							

**vmPFC**	**L**	–**6**	**54**	**3**	**6.00**	**0.000**	**1073**
**Cerebellum**	**R**	**27**	–**75**	–**33**	**5.93**	**0.000**	**97**
**MTG**	**L**	–**60**	–**36**	**0**	**5.57**	**0.001**	**420**
**Superior temporal gyrus**	**R**	**60**	**3**	–**3**	**5.09**	**0.009**	**75**
**Angular gyrus**	**L**	–**48**	–**60**	**30**	**5.06**	**0.010**	**297**
**Precentral gyrus**	**R**	**42**	–**21**	**66**	**4.88**	**0.022**	**315**
Precuneus	L	–15	–45	75	4.66	0.055	31
Precuneus	L	–12	–42	45	4.30	0.211	26
Cingulate gyrus	L	–9	–48	33	4.21	0.276	34
not labeled	L	–21	0	27	4.12	0.358	16
Supramarginal gyrus	L	–63	–24	18	4.08	0.405	31
Putamen	L	–30	–12	3	4.06	0.424	29
Cingulate gyrus		0	–9	39	4.02	0.472	18
Rolandic operculum	R	48	–21	24	3.95	0.549	51
Hippocampal formation	L	–27	–12	–15	3.80	0.726	15
Postcentral gyrus	R	33	–39	66	3.67	0.857	13
Superior temporal gyrus	R	66	–15	–3	3.57	0.928	12

*(A) Difference between subsequently remembered object-location associations and subsequently forgotten object-location associations during encoding (E_C > E_F) and (B) Difference between correctly retrieved object-location associations and forgotten object-location associations during retrieval (R_C > R_F). Clusters are reported at *p* < 0.001, uncorrected, with an extent threshold of *k* > 10 voxels. Clusters whose peak survived a threshold of p-FWE < 0.05 are highlighted in bold print. L, left; R, right; IOG, inferior occipital gyrus; vmPFC, ventromedial prefrontal cortex; IFG, inferior frontal gyrus; MTG, middle temporal gyrus*.

Successful retrieval (R_C > R_F) was associated with activations of frontal regions (ventromedial prefrontal cortex, precentral gyrus, rolandic operculum), temporal regions (hippocampus, middle, and superior temporal gyrus), parietal regions (angular gyrus, precuneus, supramarginal gyrus, postcentral gyrus), the cingulate cortex (posterior and mid cingulate), and the putamen (Table 1B and Figure 4b).

### Resting State fMRI Data

#### Step 1: Seed-to-Voxel Analyses During the Control Condition

Clusters showing an SME during encoding, i.e., the right IOG/fusiform gyrus (*x* = 45, *y* = -75, *z* = -12), the right orbitofrontal cortex (*x* = 27, *y* = 33, *z* = -12), and the left ventromedial prefrontal cortex (vmPFC) (*x* = -3, *y* = 33, *z* = -18), were defined as seeds for seed-to-voxel connectivity analyses.

In the control condition, FC increased during post-encoding rest (R3; compared to baseline, R1) between the right IOG/fusiform seed and the left MTG (*x* = -44, *y* = -28, *z* = -10; *k* = 294; *p* < 0.01, FWE-corrected), the triangular part of the left IFG (*x* = -54, *y* = 20, *z* = 26; *k* = 121; p-FWE < 0.05), as well as the orbital part of the left IFG (*x* = -48, *y* = 36, *z* = -6; *k* = 157; *p* < 0.05, FWE-corrected).

Seeds located at the right orbitofrontal cortex and the left vmPFC did not show significant changes in functional connectivity with other regions.

Furthermore, neither the reminder nor the interference condition revealed significant FC increases for the specified regions during post-encoding rest.

#### Step 2: ROI-to-ROI Analyses

To test whether the above detected connectivity changes between R1 and R3 were modulated by cognitive interference and reminders, ROI-to-ROI-analyses between the clusters resulting from the seed-to-voxel analysis within the control condition (see above) were calculated for all three experimental conditions (i.e., between the right IOG/fusiform gyrus on the one hand and the MTG, the triangular part of the left IFG as well as the orbital part of the left IFG on the other hand).

ANOVAs revealed significant interaction effects between modulation and resting state in the FC between the right IOG/fusiform gyrus and the left MTG [*F*(2,16) = 7.70, *p* < 0.05], between the right IOG/fusiform gyrus and the triangular part of the left IFG [*F*(2,16) = 4.68, *p* < 0.05], and between the right IOG/fusiform gyrus and the orbital part of the left IFG [*F*(2,16) = 6.78, *p* < 0.05]. Effects were corrected for multiple comparisons using FDR-correction.

As identified by *post hoc t*-tests, these interaction effects were due to significant increases in FC between R1 and R3 in the control condition for all ROI-combinations (right IOG/fusiform gyrus – left MTG: *t*(17) = -5.079, *p* < 0.01, Bonferroni-corrected; right IOG/fusiform gyrus – left IFGtriangular: *t*(17) = -4.730, *p* < 0.01, Bonferroni-corrected; right IOG/fusiform gyrus – left IFGorbital: *t*(17) = -4.295, *p* < 0.01, Bonferroni-corrected), as expected from the seed-to voxel analysis in step 1 (see above), but no such increases in the interference condition (right IOG/fusiform gyrus – left MTG: *t*(17) = -0.943, *p* = 0.359, Bonferroni-corrected; right IOG/fusiform gyrus – left IFGtriangular: *t*(17) = -0.142, *p* = 0.889, Bonferroni-corrected; right IOG/fusiform gyrus – left IFGorbital: *t*(17) = -1.023, *p* = 0.321, Bonferroni-corrected) or in the reminder conditions (right IOG/fusiform gyrus – left MTG: *t*(17) = -0.624, *p* = 0.541, Bonferroni-corrected; right IOG/fusiform gyrus – left IFGtriangular: *t*(17) = -0.817, *p* = 0.425, Bonferroni-corrected; right IOG/fusiform gyrus – left IFGorbital: *t*(17) = 0.381, *p* = 0.708, Bonferroni-corrected).

The nature of the interaction effects was further explored by comparing the connectivity changes between the first and the third resting state (Δ FCR3 – FCR1) across the experimental conditions:

*T*-tests revealed that the FC increase between the right IOG/fusiform gyrus and the left MTG from baseline to post-encoding rest was significantly reduced after both interference (CFCR3–FCR1: 0.249 (0.049), INFCR3–FCR1: 0.051 (0.054), *t*(17) = -2.983, *p* < 0.05) and reminders (REFCR3–FCR1: 0.035 (0.056), *t*(17) = -3.403, *p* = 0.01), when compared to the control condition.

The increase of FC between the right IOG/fusiform gyrus and the triangular part of the left IFG was significantly reduced in the interference condition when compared to the control condition [CFCR3–FCR1: 0.236 (0.050), INFCR3–FCR1: 0.007 (0.047), *t*(17) = -3.052, *p* < 0.05], and non-significantly reduced in the reminder condition [REFCR3–FCR1: 0.047 (0.057), *t*(17) = -2.386, *p* = 0.087].

Functional connectivity differences between the right IOG/fusiform gyrus and the orbital part of the left IFG were significantly reduced in the reminder condition when compared to control [CR3-R1: 0.176 (0.041), REFCR3–FCR1: -0.020 (0.053), -3.603, *p* < 0.01], and non-significantly reduced after interference [INFCR3–FCR1: 0.044 (0.043), *t*(17) = -2.410, *p* = 0.083].

Between the reminder and interference conditions, there were no FC differences between regions of interest.

**Figure 5:**
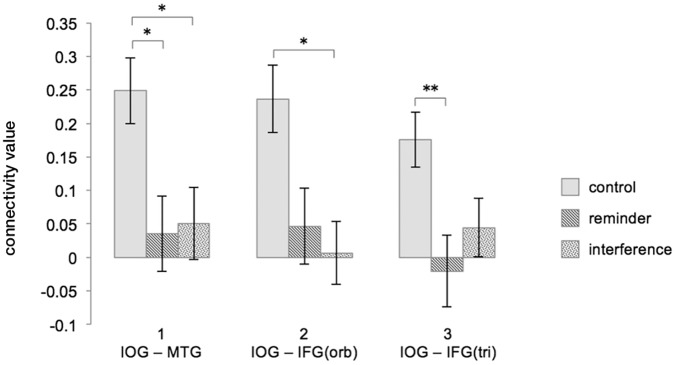
Interferences and reminders disrupt FC increase during post-encoding rest (ΔFCR3 – FCR1). Depicted are combinations of brain regions showing a significant FC increase during undisturbed post-encoding rest in the control condition and how the different modulations change FC in the ROI-to-ROI analysis. Column 1 refers to the FC between the right IOG/fusiform gyrus and the left MTG, column 2 refers to the FC between the right IOG/fusiform gyrus and the orbital part of the left IFG, and column 3 refers to the FC between the right IOG/fusiform gyrus and triangular part of the left IFG. Connectivity values represent fisher-transformed correlation coefficients. Significant differences are marked with an asterisk at *p* < 0.05 and with two asterisks at *p* < 0.01 (Bonferroni-corrected). Error bars depict the standard error. FC, functional connectivity; C, control; RE, reminder; IN, interference; IOG, inferior occipital gyrus/fusiform gyrus; MTG, middle temporal gyrus; IFG, inferior frontal gyrus; orb, orbital; tri, triangular.

Figure 5 shows an illustration of the difference scores (Δ FCR3 – FCR1) across the experimental conditions. For details of all performed *t*-tests please see Table 2.

**Table 2:** Changes in functional connectivity between R1 and R3.

(A) FC score differences between R3 and R1				
**ROI 1 (peak *x*, *y*, *z*)**	**ROI 2 (peak *x*, *y*, *z*)**	**Conditions**	**M (SE)**	

Right IOG/fusiform gyrus	left middle temporal gyrus	C	0.249 (0.049)	
(*x* = 45, *y* = -75, *z* = -12)	(*x* = -44, *y* = -28, *z* = -10)	RE	0.035 (0.056)	
		IN	0.051 (0.054)	
Right IOG/fusiform gyrus	left inferior frontal gyrus, triangular	C	0.236 (0.050)	
	(*x* = -54, *y* = 20, *z* = 26)	RE	0.047 (0.057)	
		IN	0.007 (0.047)	
Right IOG/fusiform gyrus	left inferior frontal gyrus, orbital	C	0.176 (0.041)	
	(*x* = -48, *y* = 36, *z* = -6)	RE	-0.020 (0.053)	
		IN	0.044 (0.043)	

**(B) *Post hoc t*-test results, *p*-values corrected for multiple comparisons (Bonferroni)**				

**ROI 1**	**ROI 2**	**Comparisons**	***t*(17)**	***p***

Right IOG/fusiform gyrus	Left middle temporal gyrus	C : RE	–3.403	0.010*
		C : IN	–2.983	0.025*
		RE : IN	0.197	1.000
Right IOG/fusiform gyrus	Left inferior frontal gyrus, triangular	C : RE	–2.386	0.087
		C : IN	–3.052	0.022*
		RE : IN	–0.551	1.000
Right IOG/fusiform gyrus	Left inferior frontal gyrus, orbital	C : RE	–3.603	0.007**
		C : IN	–2.410	0.083
		RE : IN	1.053	0.922

*(A) Mean differences (M) and standard errors (SE) of the differences between R3 and R1 (Δ R3-R1) of the FC between ROI 1 and ROI 2. ROI 1 was defined as a region showing a subsequent memory effect during encoding. ROI 2 represents regions that showed a significant increase in FC with ROI 1 during undisturbed consolidation. (B) Post hoc *t*-test results comparing the mean differences of FC between the three conditions (C, RE, and IN). Results are corrected for multiple comparisons using Bonferroni correction. IOG, inferior occipital gyrus; C, control; RE, reminder; IN, interference. Significant differences at *p* < 0.05 are marked with an asterisk and with two asterisks at *p* < 0.01*.

Between R1 and R2, no significant FC changes could be observed between regions of interest in all experimental conditions, which was calculated by paired *t*-tests. Results are presented in Table 3.

**Table 3:** Changes in functional connectivity between R1 and R2.

ROI pair	Conditions	M (SE)	T (df)	*p*
Right IOG/fusiform gyrus – left MTG	C	–0.085 (0.055)	–1.553 (17)	0.139


	RE	0.030 (0.070)	0.449 (17)	0.659
	IN	–0.013 (0.059)	–0.229 (17)	0.821
Right IOG/fusiform gyrus – left IFGtriangular	C	–0.058 (0.204)	–1.217 (17)	0.240


	RE	–066 (0.197)	1.423 (17)	0.173
	IN	0.056 (0.179)	1.318 (17)	0.205
Right IOG/fusiform gyrus – left IFGorbital	C	–0.054 (0.051)	–1.071 (17)	0.299


	RE	0.036 (0.058)	0.616 (17)	0.546
	IN	–0.034 (0.040)	–0.853 (17)	0.405

*Presented are the mean differences (M) and standard errors (SE) of the FC scores between regions of interest for R1 and R2 for the three experimental conditions and the results of the paired *t*-tests*.

#### FC Between Encoding and Retrieval

**Figure 6:**
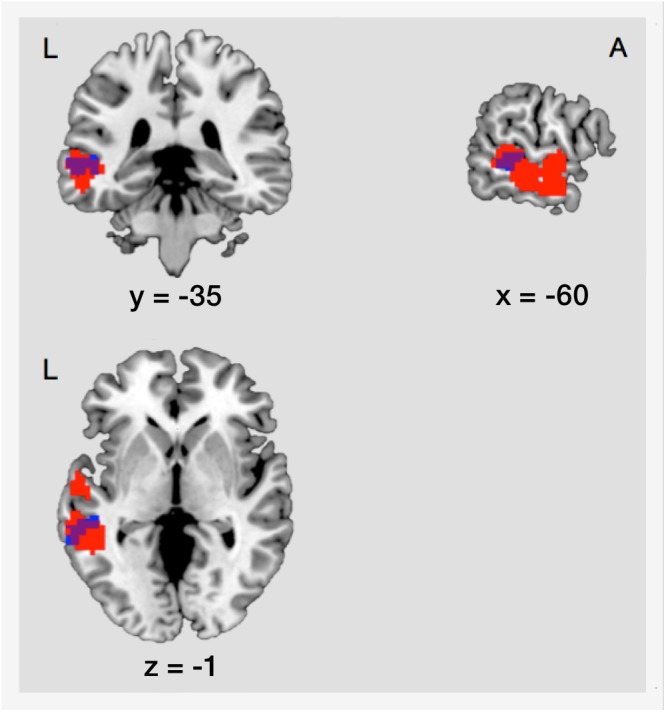
Structural overlap of regions involved in consolidation and those involved in retrieval. Blue, cluster showing increased functional connectivity between the right IOG/fusiform gyrus and the left MTG during consolidation. Red, cluster showing main effect of memory during retrieval in the left MTG. The overlap covers an area of 53 voxels. L, left; A, anterior; IOG, inferior occipital gyrus/fusiform gyrus; MTG, middle temporal gyrus.

As mentioned before, there was an increase of FC between the right IOG/fusiform gyrus and the left MTG during post-encoding rest. The localization of this effect showed an anatomical overlap with activation in the left MTG during successful retrieval of locations (18.03% of the post-encoding FC cluster cover the retrieval cluster) (illustrated in Figure 6). To determine if connectivity between encoding and retrieval related structures increased during post-encoding rest, we performed a ROI-to-ROI analysis between the right IOG/fusiform gyrus (active during encoding) and the left MTG (active during retrieval). A *t*-test on the FC values between these ROIs within the control condition revealed a significant increase of functional connectivity during late post-encoding rest (R3) compared to baseline (R1) [*t*(17) = 3.00, *p* < 0.01].

#### Relationship Between FC Changes and Memory Performance

In order to test whether FC increases between the right IOG/fusiform gyrus and the left MTG predicted retrieval performance, we performed Pearson’s correlation between the increase of FC (R3 > R1) and memory performance in the control condition. No significant association between FC increase and successful retrieval was found (*r* = 0.121, *p* = 0.633).

## Discussion

Our data indicate that during post-encoding rest functional connectivity between regions showing a SME and regions mediating memory retrieval increases. During this phase, links between encoding- and retrieval-related brain regions appear to be established. By experimental manipulation, we show that these links are initially vulnerable to cognitive interference. While both interfering and reminding stimuli disrupted connectivity, only interfering stimuli worsened memory performance. This indicates that consolidation must rely on additional processes not observed here.

### Increases of Functional Connectivity Based on Encoding Related Areas Can Be Observed During Post-encoding Rest

In a data-driven approach, we observed that functional connectivity increases during early post-encoding rest originated in regions mediating encoding, such as the IOG/fusiform gyrus. Occipito-basal regions such as the IOG and the fusiform gyrus are not only involved in the processing and analysis of incoming visual information but have repeatedly been shown to be centrally implicated in memory encoding (Cabeza et al., 1997; Morcom et al., 2003; Garoff et al., 2005), consistent with prior work using a paradigm comparable to the one used in the current study (Kukolja et al., 2016). Connectivity increases with these regions may constitute a first step in forming a memory trace, which is in line with prior work suggesting that regions mediating initial encoding also play a central role in memory consolidation (Foster and Wilson, 2006; Kukolja et al., 2016). One putative mechanism underlying increased functional connectivity is repetitive (re-)activation of memory-specific networks during unconscious replay of memories (Hoffman and McNaughton, 2002; Deuker et al., 2013; Atherton et al., 2015). Differences in FC were pronounced in R3, while these changes were not yet observable at an earlier stage of post-encoding rest. This supports the idea that functional links are not established immediately after encoding but become stronger with the passage of time. Such temporal dynamics of connectivity changes have already been observed in an earlier study (Jacobs et al., 2015).

### Functional Connectivity Increases Between Encoding- and Retrieval-Related Regions During Post-encoding Rest

During undisturbed post-encoding rest, an increase of functional connectivity between brain regions, which are crucial for encoding, and those mediating successful retrieval, could be observed, i.e., between the right IOG/fusiform gyrus and the left MTG. Recent evidence suggests that the MTG is critically involved in memory consolidation. Next to showing increased blood perfusion during post-encoding rest (Groen et al., 2011), the MTG also showed increased local intrinsic connectivity during consolidation in our prior work (Kukolja et al., 2016). Interestingly, the MTG effects in our prior work were located at the right hemisphere and more posterior than the present findings. We speculate that this in part may be due to the differences in task design, which should be focused in further studies.

A number of studies have described the involvement of the MTG in episodic memory retrieval (Nyberg et al., 1996; Spaniol et al., 2009). However, the lateral temporal cortex, which comprises the MTG, is not equally activated when memories are retrieved at different time points after encoding. Retrieval-related activity changes represent initially weaker representations for recent memories, which grow stronger with increasing time (Bonnici et al., 2012). These results corroborate specific consolidation-related dynamics in this region.

Most studies reporting left MTG activation associated it with retrieval of semantic information (see Svoboda et al., 2006 for review). The present results, however, suggest that activation of the left MTG exceeds semantic processing and is also generalizable to non-semantic memories although one might argue that successful retrieval in our study may have been linked to individual semantic strategies in order to better memorize object-location associations.

It is unlikely that the connectivity increase between the right IOG/fusiform gyrus and the left MTG represents mere memory replay since the MTG was not activated during encoding. A reactivation of cortical networks coding individual stimuli is thought to be a requirement for memory reinstatement during retrieval, as suggested by recent imaging studies often using multivoxel pattern analysis (Johnson et al., 2009; Johnson et al., 2015; Wing et al., 2015). One explanation for the observed connectivity increase with the MTG is that it primes the left MTG as a neural hub for subsequent retrieval success.

Importantly, however, connectivity increases during post-encoding rest originating in areas mediating memory encoding were not restricted to regions active during subsequent retrieval. Conversely, areas active during retrieval exceeded those targeted by connectivity increases during post-encoding rest. Together, this implies that these connectivity increases cannot be regarded as specific to linking encoding- with retrieval-related areas.

### Modulation by Interfering and Reminding Stimuli

Specificity of connectivity changes during rest is usually difficult to establish since activity cannot be linked to specific events or trials. To overcome this problem, we introduced experimental manipulations that served to challenge our hypotheses. Importantly, we were able to modify connectivity changes during post-encoding rest by experimental manipulation: Presenting interfering information prevented increases in connectivity between the right IOG/fusiform gyrus and the left MTG as well as between the right IOG/fusiform gyrus and the triangular and the orbital part of left IFG, associated with reduced memory performance.

The presentation of reminders similarly hindered the increase of connectivity between the right IOG/fusiform gyrus and the left MTG as well as between the right IOG/fusiform gyrus and the left IFG. Here, however, no behavioral effect was observed. We speculate that interfering and reminding stimuli share a common effect on early memory trace formation. Specifically, memory traces during early consolidation are believed to be unstable and can easily be disrupted by perception of new stimuli. Thereby, storage of novel information can still be modified and prioritized anew, eventually enabling adaptive behavior in response to changing environments (Alberini and LeDoux, 2013).

The crucial difference between interfering and reminding stimuli, as reflected in a different behavioral outcome, was that interfering stimuli contained information incongruent to the initially encoded information. This novel information partially replaced previously encoded information and resulted in a poorer memory for the initially learned material.

Reminders, lacking such new information, likely served as reactivators of previously learned object-location associations. This is supported by numerically (albeit not significantly) increased memory performance in the reminder condition. The observed FC disruption is in line with recent evidence showing that even consolidated memories can be destabilized again by reactivation, transferring memory traces into a labile state again where they become sensitive for disruption and modification (Dudai, 2006; Nader and Hardt, 2009; Alberini and LeDoux, 2013).

An alternative but less convincing explanation is that reminders actually acted as another form of interference, causing FC disruption. More precisely, it could be argued that the neutral location, where reminders appeared, also contained information incongruent with the initially learned object-location association and thereby interfered with memory formation. However, the numerically even better performance during the RE than during the control condition renders this interpretation less plausible.

Importantly, by introducing a low-level attention task during the control condition we ensured that episodic memory but not working memory was measured and manipulated. Furthermore, it is unlikely that the observed connectivity changes between baseline and post-encoding rest and between the three experimental conditions were due to different levels of tiredness, since we included fatigue scores in our statistical models and since fatigue scores were not different between conditions.

### Limitations

A limitation of the study is that we could not detect a significant relationship between FC changes during post-encoding rest and memory performance, which challenges the interpretation that FC changes were specific to consolidation. The lack of correlation between connectivity changes and behavior might be due to little variance in memory performance within the examined sample since the study sample consisted of healthy young participants. We expect to find a clearer link between consolidation-related FC changes and memory performance by increasing behavioral variance. For this purpose, future projects will introduce the same paradigm to a sample with higher age and relatively reduced memory performance.

However, both the IN and the RE interventions clearly interrupted FC increases, suggesting that this increase was related to undisturbed consolidation.

An alternative explanation is that the changes in connectivity observed in the present study were not related to consolidation processes but may rather reflect residuals of preceding tasks. One can argue, however, that any residuals in neural activity following an encoding task at least in part contains activity mediating consolidation. The modulation of post-encoding connectivity by interfering tasks supports this interpretation. In order to clarify this issue, studies with patients suffering from encoding deficits are needed.

## Conclusion

The present findings suggest that during post-encoding rest, functional links between a subset of brain regions relevant for memory encoding and a subset of those mediating subsequent retrieval are formed.

Experimental modulation of the observed FC changes by use of interfering and reminding stimuli showed that initial connectivity increases were unstable during early phases of post-encoding rest, possibly enabling experience-based adaptive memory changes.

Since these imaging results were not corroborated by behavioral data, further studies with alternative designs are needed to verify the interpretations of the present results.

## Ethics Statement

This study was carried out in accordance with the recommendations of the good scientific practice guidelines of the University of Cologne. The protocol was approved by the ethics committee of the Faculty of Medicine, University of Cologne. All subjects gave written informed consent in accordance with the Declaration of Helsinki.

## Author Contributions

OR and JK planned the concept and the design of the study. OR recruited the participants, performed the measurements and calculations, and wrote the manuscript. JK, JD, OO, BvR, and NR assisted with measurements and calculations and contributed to the manuscript. JK and GF provided funding and edited the manuscript.

## Conflict of Interest Statement

The authors declare that the research was conducted in the absence of any commercial or financial relationships that could be construed as a potential conflict of interest.

## Footnotes

1 http://rfmri.org/DPARSF

2 http://www.fil.ion.ucl.ac.uk/spm

3 http://rfmri.org/DPABI

4 http://www.nitrc.org/projects/conn
